# Development of a prediction model for breast cancer based on the national cancer registry in Taiwan

**DOI:** 10.1186/s13058-019-1172-6

**Published:** 2019-08-13

**Authors:** Ching-Chieh Huang, Soa-Yu Chan, Wen-Chung Lee, Chun-Ju Chiang, Tzu-Pin Lu, Skye Hung-Chun Cheng

**Affiliations:** 1 Institute of Epidemiology and Preventive Medicine, Department of Public Health, College of Public Health, National Taiwan University, Taipei, Taiwan; 2 Department of Computing and Information, Koo Foundation Sun-Yat Sen Cancer Center, Taipei, Taiwan; 3 Taiwan Cancer Registry, Taipei, Taiwan; 4 Department of Surgery, National Taiwan University Hospital, Taipei, 100 Taiwan; 5 Department of Radiation Oncology, Koo Foundation Sun Yat-Sen Cancer Center, No. 125, Lih-Der Road, Pei-Tou District, Taipei, Taiwan

**Keywords:** Breast cancer, Prognosis, Asian, Taiwan Cancer Registry, SEER, Survival, Mortality, Model

## Abstract

**Background:**

This study aimed to develop a prognostic model to predict the breast cancer-specific survival and overall survival for breast cancer patients in Asia and to demonstrate a significant difference in clinical outcomes between Asian and non-Asian patients.

**Methods:**

We developed our prognostic models by applying a multivariate Cox proportional hazards model to Taiwan Cancer Registry (TCR) data. A data-splitting strategy was used for internal validation, and a multivariable fractional polynomial approach was adopted for prognostic continuous variables. Subjects who were Asian, black, or white in the US-based Surveillance, Epidemiology, and End Results (SEER) database were analyzed for external validation. Model discrimination and calibration were evaluated in both internal and external datasets.

**Results:**

In the internal validation, both training data and testing data calibrated well and generated good area under the ROC curves (AUC; 0.865 in training data and 0.846 in testing data). In the external validation, although the AUC values were larger than 0.85 in all populations, a lack of model calibration in non-Asian groups revealed that racial differences had a significant impact on the prediction of breast cancer mortality. For the calibration of breast cancer-specific mortality, *P* values < 0.001 at 1 year and 0.018 at 4 years in whites, and *P* values ≤ 0.001 at 1 and 2 years and 0.032 at 3 years in blacks, indicated that there were significant differences (*P* value < 0.05) between the predicted mortality and the observed mortality. Our model generally underestimated the mortality of the black population. In the white population, our model underestimated mortality at 1 year and overestimated it at 4 years. And in the Asian population, all *P* values > 0.05, indicating predicted mortality and actual mortality at 1 to 4 years were consistent.

**Conclusions:**

We developed and validated a pioneering prognostic model that especially benefits breast cancer patients in Asia. This study can serve as an important reference for breast cancer prediction in the future.

**Electronic supplementary material:**

The online version of this article (10.1186/s13058-019-1172-6) contains supplementary material, which is available to authorized users.

## Background

Globally, breast cancer is the most common incident cancer. In 2017, cancer was the leading cause of death in Taiwan, and this has been the case for the past 36 years as well as the leading cause of cancer deaths and disability-adjusted life years in women [1]. Interestingly, Asians constitute the largest proportion of breast cancer patients worldwide [2]. Consequently, it is essential to assess prognostic risk factors, treatment effects, and survival rates in women with breast cancer in Taiwan or, more broadly, in Asia.

In recent years, several research studies have been performed to predict the survival rates of breast cancer patients. For example, PREDICT [3–5] is a useful prognostic algorithm implemented online to predict the breast cancer-specific survival and overall survival of female patients with early-stage breast cancer in Britain. This web-based tool can help breast cancer patients and physicians to estimate survival rates in the next few years, as well as predict treatment effects. However, most of the prognostic models for breast cancer patients focus only on Western populations, and previous studies indicate that breast cancer survival rates vary greatly between Asian and European people, due not only to treatment or environmental factors, but also to fundamental genetic variation [6–9].

In light of this concern, the first aim of this study was to develop a prognostic model to predict the overall survival and breast cancer-specific survival in Taiwan. Based on the results, we are able to predict the mortality of breast cancer patients in Asia. The data used for model development were from a large cohort followed by the Taiwan Cancer Registry (TCR) and included systemic treatments, site-specific factors, and long-term outcome tracking in 20 hospitals nationwide from 2011 to 2015. The second aim of this study was to assess the impact of racial differences based on model validation in different populations. We verified our model through the model discrimination and calibration against the Asian, white, and black populations in the US-based Surveillance, Epidemiology, and End Results (SEER) database [10].

## Methods

### Data source and sample selection

**Fig. 1:**
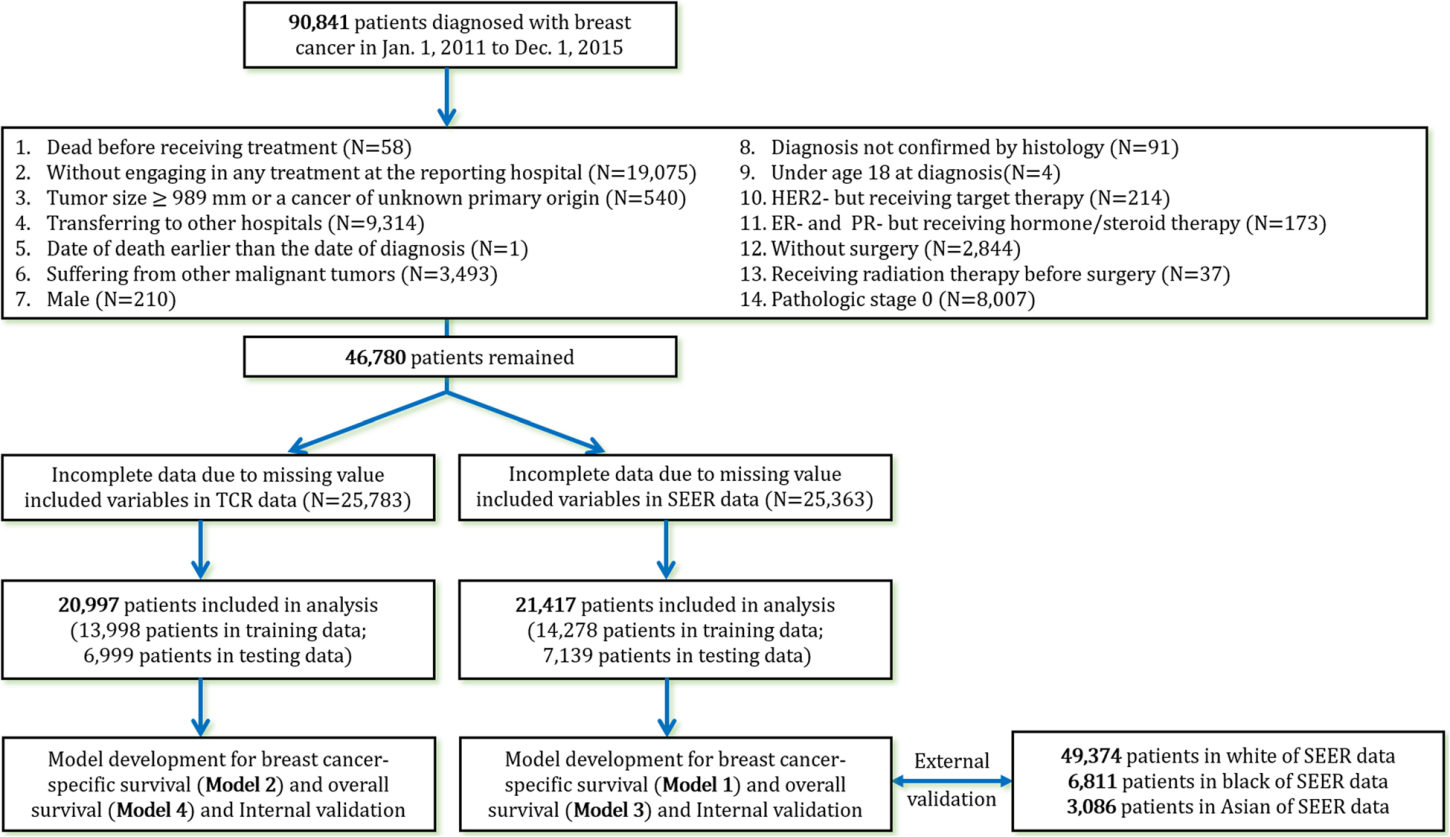
Flow diagram of exclusion criteria and statistical analysis

The original data for our primary analysis were retrieved from the TCR database. The TCR provides complete core information for cancer cases in Taiwan that meet the criteria for high data quality [11, 12]. Its data source consists of 19 medical centers and a district hospital specializing in cancer research, Koo Foundation Sun Yat-Sen Cancer Center. A total of 90,841 patients diagnosed with breast cancer from January 1, 2011, to December 31, 2015, were recruited, and the last follow-up date was December 31, 2017. The study flow is illustrated in Fig. 1.

The exclusion criteria included death before receiving treatment, not engaging in any treatment at the reporting hospital, having tumor size ≥ 989 mm or a cancer of unknown primary origin, transferring to other hospitals, suffering from other malignant tumors, receiving radiation therapy before surgery, and being male. Also, excluded were cases with pathologic stage 0; patients whose date of death was earlier than the date of diagnosis; cases without surgery; patients whose cancer genotype was human epidermal growth factor receptor 2-negative (HER2−) but who still received targeted therapy; patients with hormone receptor-negative (HR−) cancer, i.e., estrogen receptor-negative (ER−) and progesterone receptor-negative (PR−), who received hormone/steroid therapy; cases with a diagnosis not confirmed by histology; and patients under age 18 at diagnosis. The number of patients excluded for each reason is presented in Fig. 1. The ER+ and PR+ genotypes were defined as more than one tumor specimen, as the definition in SEER Registrar Staging Assistant (SEER*RSA). HER2+ status was required to be established by immunohistochemistry, chromogenic in situ hybridization, fluorescence in situ hybridization, or similar methods.

### Prognostic model development and internal validation

We considered overall survival and breast cancer-specific survival as study endpoints. A Cox proportional hazards model was adopted in R using the package “survival” [13] to estimate the hazard ratio associated with various risk factors or clinical treatments. Chemotherapy, radiation therapy, hormone/steroid therapy, and targeted therapy were treated as binary variables, expressed as yes/no answers to the question, “Did the patient receive this therapy?” On the other hand, age at diagnosis, tumor size, and the node-positive ratio (regional lymph nodes positive/regional lymph nodes examined) were continuous variables. Tumor grade, lymph vessel or vascular invasion, breast cancer subtype, and pathologic stage were categorical variables. Breast cancer subtypes were categorized as proposed by Blows [12] and Cheng [13], with grouping into three major subtypes: (1) luminal-like subtype (HR+ and HER2−), (2) HER2 subtype (HER2+), and (3) triple-negative subtype (HR− and HER2−). For tumor grade, 1 means the well differentiated or differentiated; 2 means moderately differentiated, moderately well differentiated, or intermediate differentiation; and 3 means poorly differentiated, dedifferentiated, undifferentiated, or anaplastic. Lymph vessel or vascular invasion defined “Yes” as having lymph vessel or vascular invasion and defined “No” as otherwise.

We used a data-splitting strategy, first randomly separating the TCR data into training and testing datasets with the ratio 2:1 [14]. The training data were used to construct our prognostic model and to estimate the variable coefficients; the testing data were analyzed for internal validation. In addition, one previous report demonstrated that the effect of the continuous variables, e.g., age at diagnosis and tumor size, was not linear [4]. Therefore, we used the following methods to deal with this issue.

A multivariable fractional polynomial (MFP) approach is commonly adopted in medical research [4, 15, 16] to determine the importance of variables and their functional forms (nonlinear forms) for model development. Among the prognostic variables that we considered, age at diagnosis, tumor size, and the node-positive ratio were continuous variables that were further transformed by the MFP approach. The MFP approach was carried out in R using the package “mfp” [17]. Because the SEER data did not include information on hormone/steroid therapy, targeted therapy, and lymphatic vessel or vascular invasion, we developed another prognostic model which contained all prognostic factors for overall and the breast cancer-specific survival.

### External model validation

Our model was validated against the white, the black, and the Asian subpopulations in the SEER database. The follow-up cutoff date of the SEER data was December 31, 2015, and the diagnosis year was from 2011 to 2015. The definition of “Asian” in this study included Chinese, Japanese, Korean, Vietnamese, and Laotian ethnicity. We again emphasize that the model validation based on the SEER data could only be implemented using prognostic factors that were also available in SEER data for both overall survival and breast cancer-specific survival (model 1 and model 3 presented in Fig. 1).

Model discrimination and calibration were evaluated separately in our study. For discrimination, the area under the receiver operating characteristic curve (AUC) was assessed. The concordance probability was one primary indicator to assess the discriminatory power and to predict the AUC of a Cox model. Harrell’s c-index of concordance [18] was utilized as an alternative measure of discrimination. Harrell’s c-index expresses the probability that the predicted event and the observed event (i.e., survival) are consistent based on informative pairs. A Harrell’s c-index ≤ 0.5 indicates a poor model, > 0.7 indicates a good model, and > 0.8 indicates a strong model.

Model calibration was also evaluated to explore whether there were significant differences (*P* value < 0.05) between the predicted mortality and the observed mortality in total or in each category by a given follow-up time after diagnosis. The predicted mortality for cases was calculated from the coefficient estimated by the Cox model [4, 14, 16].

## Results

### Prognostic model development

We summarize the MFP function of continuous variables and the associated logarithmic hazard ratios (logHRs) for breast cancer-specific survival regressed on variables contained in the SEER database (model 1) in Table 1. As mentioned earlier, the SEER database did not contain all prognostic factors present within the TCR. An additional model for the breast cancer-specific survival containing all the important variables in the TCR (model 2) is presented in Table 2. The estimated coefficients in Tables 1 and 2 are all in line with observations from clinical practice. Conducting chemotherapy (logHR = − 0.4792 in Table 1 and − 0.4147 in Table 2), hormone/steroid therapy (logHR = − 0.8397 in Table 2), targeted therapy (logHR = − 0.4687 in Table 2), or radiation therapy (logHR = − 0.3316 in Table 1 and − 0.3203 in Table 2) does indeed improve the survival of breast cancer patients. In terms of the breast cancer subtypes, patients with the triple-negative subtype have a higher risk of mortality (logHR = 1.426 in Table 1 and 0.6457 in Table 2) than the others, as is expected. In addition, overall survival model containing variables in the SEER database (model 3) is shown in Additional file 1: Table S1, and overall survival model containing all variables (model 4) is presented in Additional file 1: Table S2.

**Table 1:** Multivariable fractional polynomial functions and logarithm hazard ratios for breast cancer-specific survival using SEER variables

Variables	Function	logHR	*P* value
Continuous variables			
Age1	(age/100)^−0.5^	− 13.05	< 0.001
Age2	(age/100)^−0.5^ × log(age/100)	10.42	< 0.001
Tumor size, mm	log(size/10)	0.8375	< 0.001
Node-positive ratio	((ratio + 0.1)/0.1)^0.5^	0.7076	< 0.001
Categorical variables			
Chemotherapy			
Without	–	–	–
With	–	− 0.4792	< 0.001
Radiotherapy			
Without	–	–	–
With	–	− 0.3316	< 0.001
Grade			
1	–	–	–
2	–	0.6106	0.003
3	–	1.059	< 0.001
Subtype			
Luminal-like	–	–	–
HER2	–	0.3185	< 0.001
Triple negative	–	1.426	< 0.001
Pathological stage			
1	–	–	–
2	–	0.6073	< 0.001
3	–	1.076	< 0.001
4	–	2.044	< 0.001

Derived from model 1

**Table 2:** Multivariable fractional polynomial functions and logarithm hazard ratios for breast cancer-specific survival using all variables

Variables	Function	logHR	*P* value
Continuous variables			
Age1	(age/100)^−0.5^	− 10.87	0.0035
Age2	(age/100)^−0.5^ × log(age/100)	8.968	< 0.001
Tumor size, mm	log(size/10)	0.7678	< 0.001
Node-positive ratio	((ratio + 0.1)/0.1)^0.5^	0.5339	< 0.001
Categorical variables			
Chemotherapy			
Without	**–**	–	–
With	–	− 0.4147	< 0.001
Radiotherapy			
Without	–	–	–
With	–	− 0.3203	< 0.001
Grade			
1	–	–	–
2	–	0.4795	0.015
3	–	0.818	< 0.001
Subtype			
Luminal-like	–	–	–
HER2	–	0.1806	0.22
Triple negative	–	0.6457	< 0.001
Pathological stage			
1	–	–	–
2	–	0.5311	< 0.001
3	–	1.134	< 0.001
4	–	2.172	< 0.001
Targeted therapy			
Without	–	–	–
With	–	− 0.4687	0.001
Hormone/steroid therapy			
Without	–	–	–
With	–	− 0.8397	< 0.001
Lymph vessel or vascular invasion (LVI)			
No	–	–	–
Yes	–	0.3321	< 0.001

Derived from model 2

Cumulative baseline hazards were also formulated by the MFP approach. The non-linear transformation for the time (days) after diagnosis of the four models is the same and contained two terms:

time1 = (time/1000)^2^ and time2 = log (time/1000)(time/1000)^2^.

The corresponding time-dependent cumulative baseline hazard functions (CBH_t_) for models 1 to 4 were as follows:

$$ \mathrm{CBHt}\ \left(\mathrm{model}\ 1\right)=-0.001446+0.0121\times \mathrm{time}1-0.006314\times \mathrm{time}2 $$

$$ \mathrm{CBHt}\ \left(\mathrm{model}\ 2\right)=-0.001237+0.01215\times \mathrm{time}1-0.006522\times \mathrm{time}2 $$

$$ \mathrm{CBHt}\ \left(\mathrm{model}\ 3\right)=-0.00168+0.02064\times \mathrm{time}1-0.009897\times \mathrm{time}2 $$

$$ \mathrm{CBHt}\ \left(\mathrm{model}\ 4\right)=-0.001563+0.02077\times \mathrm{time}1-0.01028\times \mathrm{time}2 $$

**Fig. 2:**
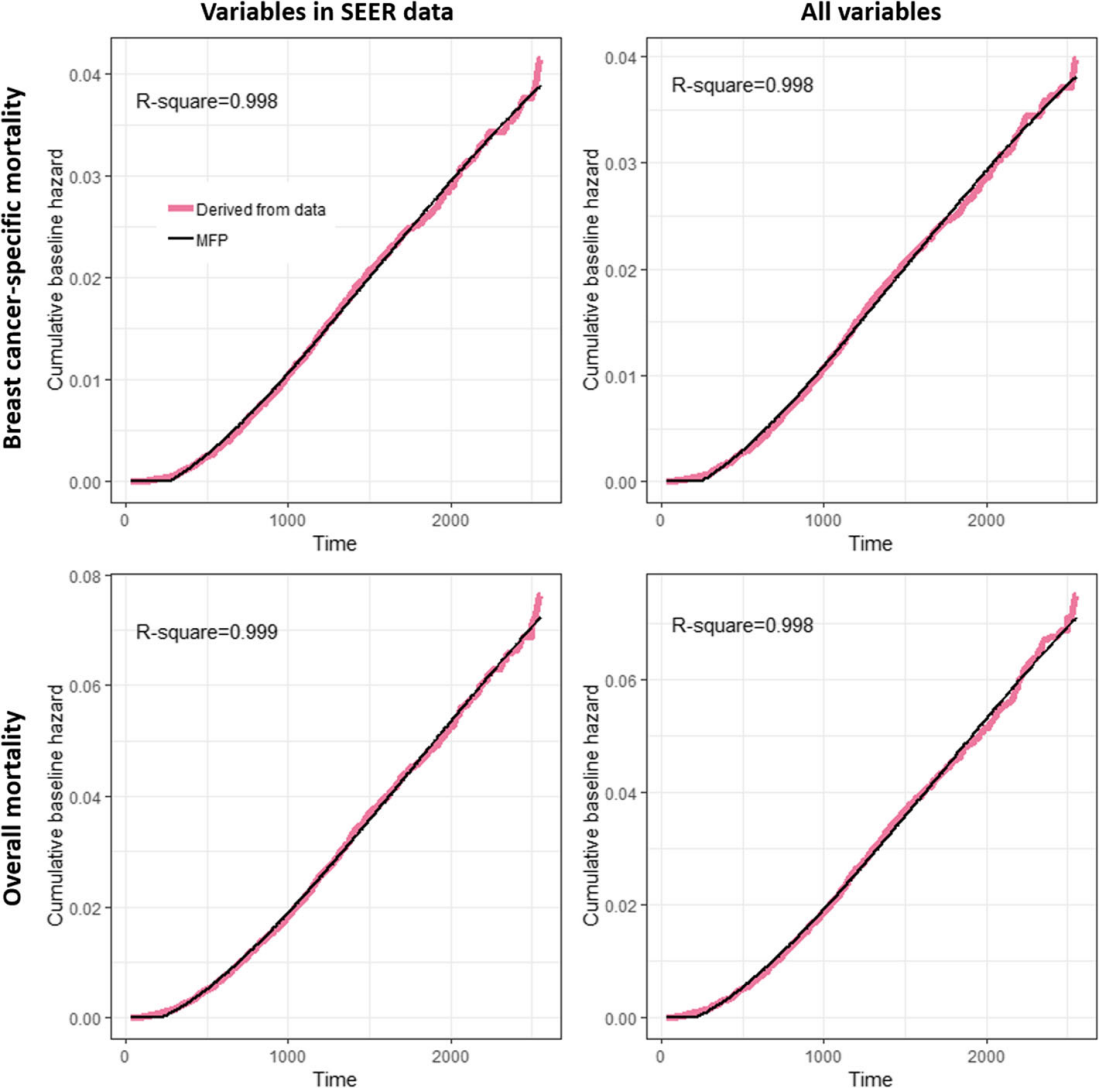
Cumulative baseline hazard derived from data and the MFP estimation

If the prediction of cumulative baseline hazard was less than 0, it was replaced with 0. The four functions fitted the corresponding cumulative baseline hazard well. As shown in Fig. 2, the R-square values were all nearly equal to 1. Then the survival estimation $\hat{S}\left(t|\mathbf{Z},\boldsymbol{\upbeta} \right)$ given the time *t* and prognostic variables **Z** with corresponding logHR **β** can be calculated by exp (−CBH_t_ × **Z**^*T*^ **β**).

### Model discrimination and calibration

In follow-up analyses, the Harrell’s c-indices were all higher than 0.8, indicating good discrimination ability across all data (Table 3). However, a significant difference between predicted mortality and observed mortality in calibration was observed for non-Asian groups (Table 4), indicating that our model generally underestimated the mortality of the black population, and in the white population, our model underestimated the mortality at 1 year post-diagnosis and overestimated it at 4 years post-diagnosis. These results show that the estimated risk of death in our TCR-based model is not useful for application in individuals with non-Asian ethnicity [19]. Although the Harrell’s c-indices performed well, suggesting certain prognostic variables remained important for non-Asian patients, the estimation of life expectancy is incorrect due to the low calibration value, especially in the black population [19]. In terms of the Asian population in Table 4, although mortality of model 3 calibration at 1 year post-diagnosis for Asians in the SEER database is slightly underestimated, the mortality of model 1 is calibrated well in general. In terms of the training and testing data in Table 4 or Additional file 1: Table S3, all *P* values > 0.05, indicating there was no significant difference between predicted mortalities and observed mortalities.

**Table 3:** Comparison of discrimination (AUCs) by the estimation of Harrell’s c-index

	Overall survival	Breast cancer-specific survival
Variables in SEER data		
Training	0.827	0.865
Testing	0.812	0.846
White	0.812	0.882
Black	0.806	0.858
Asian	0.873	0.92
All variables		
Training	0.831	0.869
Testing	0.816	0.854

The first five rows were calculated using prognostic variables in SEER data, and the last two rows were calculated using all prognostic variables

**Table 4:** Model calibration of breast cancer-specific mortality regressed on prognostic variables in SEER data

	Breast cancer-specific survival			Overall survival		
Calibration year	Observed	Predicted	*P* value	Observed	Predicted	*P* value
Training data						
1	51	52.64	0.821	83	83.87	0.924
2	229	236.75	0.615	316	317.83	0.918
3	363	373.45	0.589	481	475.71	0.808
4	425	422.94	0.920	569	544.60	0.296
5	348	350.04	0.913	466	456.30	0.650
6	220	215.38	0.753	290	275.41	0.379
Testing data						
1	33	25.28	0.125	47	41.21	0.367
2	109	114.89	0.583	142	157.12	0.228
3	177	182.81	0.668	237	237.48	0.975
4	202	206.35	0.762	266	270.31	0.793
5	176	172.28	0.777	227	226.75	0.987
6	96	104.32	0.416	129	133.50	0.697
White						
1	236	132.35	< 0.001	416	245.11	< 0.001
2	472	432.07	0.055	805	666.75	< 0.001
3	554	570.83	0.481	905	834.99	0.015
4	378	426.84	0.018	617	628.31	0.652
5	–	–	–	–	–	–
6	–	–	–	–	–	–
Black						
1	47	29.32	0.001	87	43.98	< 0.001
2	132	90.05	< 0.001	194	115.66	< 0.001
3	138	115.04	0.032	195	141.00	< 0.001
4	99	86.07	0.164	146	106.57	< 0.001
5	–	–	–	–	–	–
6	–	–	–	–	–	–
Asian						
1	10	7.57	0.377	23	13.93	0.015
2	22	24.51	0.612	43	38.03	0.421
3	21	29.84	0.106	43	44.16	0.862
4	17	23.31	0.191	29	33.42	0.445
5	–	–	–	–	–	–
6	–	–	–	–	**–**	–

Model calibration within the strata of categorical variables is presented in Table 5 and Additional file 1: Tables S4-S20). We calibrated at 3 years for model 1 (Table 5, Additional file 1: Tables S6-S7) and calibrated for 3 years for model 2 (Additional file 1: Tables S15-S16), in which age at diagnosis and tumor size were also grouped into categories by referring to the classification of Candido [4]. Additional calibration results for models 1 and 2 at 5 years are shown in Additional file 1: Tables S4-S5 and S13-S14, respectively, as are the calibration results for model 3 (Additional file 1: Tables S8-S12) and model 4 (Additional file 1: Tables S17-S20).

**Table 5:** Model 1 calibration at 3 years in the Asian population of the SEER database

	Number of cases	Observed events	Predicted events	Difference (%)	*P* value
Subtype					
Luminal-like	854	13	15.03	− 0.15	0.6
HER2	181	1	5.38	− 4.38	0.059
Triple negative	110	7	9.42	− 0.34	0.431
Grade					
1	261	0	1.52	-Inf.	0.218
2	538	4	11.55	− 1.89	0.026
3	346	17	16.76	0.013	0.954
Pathological stage					
1	577	0	3.04	-Inf.	0.081
2	457	7	14.35	− 1.05	0.052
3	102	11	9.45	0.14	0.164
4	9	3	2.98	0.003	0.995
Chemotherapy					
Without	630	4	9.62	− 1.4	0.07
With	515	17	20.21	− 0.19	0.474
Radiotherapy					
Without	397	10	11.15	− 0.11	0.73
With	748	11	18.68	− 0.7	0.075
Age at diagnosis					
< 35	17	1	0.53	0.46	0.522
35 to 49	333	7	7.54	− 0.07	0.844
50 to 64	428	7	10.8	− 0.54	0.247
65 to 74	206	3	5.59	− 0.86	0.272
75+	161	3	5.37	− 0.79	0.306
Tumor size					
< 10	260	0	0.9	-Inf.	0.341
10 to 19	388	1	4.57	− 3.57	0.095
20 to 29	257	5	6.73	− 0.34	0.505
30 to 49	161	10	8.72	0.13	0.665
50+	79	5	8.92	− 0.78	0.19

For the calibration within each categorical variable at 3 years or 5 years post-diagnosis, although there are some *P* values < 0.05 in the training data (triple-negative subtype in Additional file 1: Tables S6, S10, S13; hormone therapy in Additional file 1: Table S15), the *P* values of these variables are not significant in the testing data, indicating that they might be “false positives,” that is, the significance occurred randomly. The model 1 calibration for Asians in the SEER database (Table 5) shows that the breast cancer-specific mortality of grade 2 was slightly overestimated (4 observed versus 12 predicted events, *P* = 0.026). However, overall, the predicted and observed mortalities are approximately consistent.

## Discussion

We have developed a prognostic model to predict overall survival and breast cancer-specific survival in women with breast cancer based on the data collated from the TCR. Since external validation can further strengthen the potential applicability of a prognostic model [14, 16], we have validated our model using the US-based SEER database. A sophisticated approach, MFP, was adopted in our Cox proportional hazards model to adjust the continuous variables (age at diagnosis, tumor size, and node-positive ratio) in a non-linear functional form.

Previous studies have demonstrated that the survival rates of breast cancer patients were different in patients with distinct ethnic background [20, 21]. Hence, the applicability of our model to different races was a pivotal issue to address in our study. The discrimination seemed to perform well in all races. However, in white and black patients, the model was poorly calibrated across different years. These results reveal that non-Asian groups should be evaluated carefully in individual cases. Possible alternative solutions involve either recalibration using local data or developing a new model based on non-Asian data [19]. However, PREDICT seems to be a good choice for the non-Asian population.

In addition to the lack of calibration to some racial groups, the current model has some other limitations. Firstly, sequences between surgery and other treatments are not well defined in either the SEER database or the TCR. The sequences between treatments might cause bias. Furthermore, many risk factors related to lifestyle should also be considered in the future. Lastly, it is important to emphasize that the period of follow-up, the years since diagnosis, in our data is not sufficient and will limit our interpretations. It has been recommended that a calibration at 5 to 10 years post-diagnosis [3, 4] would more accurately reflect the survival of patients. In spite of these limitations, this study used the largest number of breast cancer samples in Asia and will have great benefits for breast cancer prediction in the Asian population. The findings from our study are intriguing enough to prompt further research on prognostic models for Asian populations, as well as further research on breast cancer-related racial differences.

## Conclusions

In summary, this study demonstrated that a new prognostic model could be practically implemented and provided results that have advanced the field of breast cancer research. We designed a powerful model that distinguishes the effects of ethnicity on survival rates in breast cancer and that accurately predicts mortality in Asians based on a large cohort of Asian breast cancer patients. We hope that this study will pave the way for new research that will benefit breast cancer patients around the world.

## Additional file

Additional file 1: Validation of models 1–4. A series of 20 tables showing the results of the model 3 and the model 4 about their details of development and calibrations. (DOCX 115 kb)

## Data Availability

The datasets supporting the conclusions of this article are included within the article and its additional files.

## Abbreviations

AUC: Area under the receiver operating characteristic curve
CBH: Cumulative baseline hazard
ER−: Estrogen receptor-negative
HER2−: Human epidermal growth factor receptor 2-negative
HR−: Hormone receptor-negative
logHR: Logarithmic hazard ratio
MFP: Multivariable fractional polynomial
PR−: Progesterone receptor-negative
SEER: Surveillance, Epidemiology, and End Results
TCR: Taiwan Cancer Registry

## References

[1] Deaths from cancer hit new high in Taiwan (update). 2018. http://focustaiwan.tw/news/asoc/201806150009.aspx.

[2] Fitzmaurice C, Akinyemiju TF, Al Lami FH, Alam T, Alizadeh-Navaei R, Allen C (2018). Global, regional, and national cancer incidence, mortality, years of life lost, years lived with disability, and disability-adjusted life-years for 29 cancer groups, 1990 to 2016: a systematic analysis for the global burden of disease study. JAMA Oncol.

[3] Wishart GC, Azzato EM, Greenberg DC, Rashbass J, Kearins O, Lawrence G, Caldas C (2010). PREDICT: a new UK prognostic model that predicts survival following surgery for invasive breast cancer. Breast Cancer Res.

[4] Candido Dos Reis FJ, Wishart GC, Dicks EM, Greenberg D, Rashbass J, Schmidt MK (2017). An updated PREDICT breast cancer prognostication and treatment benefit prediction model with independent validation. Breast Cancer Res.

[5] Wishart G, Bajdik C, Dicks E, Provenzano E, Schmidt M, Sherman M (2012). Development and validation of a prognostic model for early breast cancer that includes HER2. Br J Cancer.

[6] Leong SP, Shen Z-Z, Liu T-J, Agarwal G, Tajima T, Paik N-S, Sandelin K (2010). Is breast cancer the same disease in Asian and Western countries?. World J Surg.

[7] de Bruin MA, Kwong A, Goldstein BA, Lipson JA, Ikeda DM, McPherson L (2012). Breast cancer risk factors differ between Asian and white women with BRCA1/2 mutations. Familial Cancer.

[8] Jing L, Su L, Ring BZ (2014). Ethnic background and genetic variation in the evaluation of cancer risk: a systematic review. PLoS One.

[9] Fejerman L, Ziv E (2008). Population differences in breast cancer severity.

[10] Surveillance, Epidemiology, and End Results (SEER) Program Research Data (1973-2015), National Cancer Institute, DCCPS, Surveillance Research Program, released April 2018, based on the November 2017 submission. http://www.seer.cancer.go. Accessed Nov 2018.

[11] Chiang C-J, You S-L, Chen C-J, Yang Y-W, Lo W-C, Lai M-S (2015). Quality assessment and improvement of nationwide cancer registration system in Taiwan: a review. Jpn J Clin Oncol.

[12] Cheng C-Y, Chiang C-J, Hsieh C-H, Chang Y-K, Lai M-S (2018). Is quality of registry treatment data related to registrar experience and workload? A study of Taiwan cancer registry data. J Formos Med Assoc.

[13] Therneau TM, Lumley T (2014). Package ‘survival’. Survival analysis Published on CRAN.

[14] Altman DG, Vergouwe Y, Royston P, Moons KG (2009). Prognosis and prognostic research: validating a prognostic model. BMJ.

[15] Royston P, Ambler G, Sauerbrei W (1999). The use of fractional polynomials to model continuous risk variables in epidemiology. Int J Epidemiol.

[16] Royston P, Moons KG, Altman DG, Vergouwe Y (2009). Prognosis and prognostic research: developing a prognostic model. BMJ.

[17] Ambler G, Benner A (2010). mfp: Multivariable Fractional Polynomials. R package version 1.4. 9.

[18] Harrell FE, Lee KL, Mark DB (1996). Multivariable prognostic models: issues in developing models, evaluating assumptions and adequacy, and measuring and reducing errors. Stat Med.

[19] Matheny ME, Ohno-Machado L, Resnic FS (2005). Discrimination and calibration of mortality risk prediction models in interventional cardiology. J Biomed Inform.

[20] Curtis E, Quale C, Haggstrom D, Smith-Bindman R (2008). Racial and ethnic differences in breast cancer survival: how much is explained by screening, tumor severity, biology, treatment, comorbidities, and demographics?. Cancer.

[21] Maskarinec G, Sen C, Koga K, Conroy SM (2011). Ethnic differences in breast cancer survival: status and determinants. Women’s Health.

