# Supplementary material for: Development of a prediction model for breast cancer based on the national cancer registry in Taiwan
Source: Breast Cancer Res. 2019 Aug 13;21:92. doi: 10.1186/s13058-019-1172-6 (PMC6691540; doi:10.1186/s13058-019-1172-6)
Supplement: Supplementary file 1 — Validation of models 1–4. A series of 20 tables showing the results of the model 3 and the model 4 about their details of development and calibrations. (DOCX 115 kb) [file 13058_2019_1172_MOESM1_ESM.docx]

**Development of a prediction model for breast cancer based on the national cancer registry in Taiwan**

Ching-Chieh Huang^1^, Soa-Yu Chan^2^, Wen-Chung Lee^1,3^, Chun-Ju Chiang^1,3^, Tzu-Pin Lu^1,4^*, Skye Hung-Chun Cheng^5*^

^1^ Institute of Epidemiology and Preventive Medicine, Department of Public Health, College of Public Health, National Taiwan University, Taipei, Taiwan

^2^ Department of Computing and Information, Koo Foundation Sun-Yat Sen Cancer Center, Taipei, Taiwan

^3^ Taiwan Cancer Registry, Taipei, Taiwan

^4^ Department of Surgery, National Taiwan University Hospital, Room 501, No. 17, Xu-Zhou Road, Taipei 100, Taiwan.

^5^ Department of Radiation Oncology, Koo Foundation Sun Yat-Sen Cancer Center, Taipei, Taiwan

**Table S1.** **Multivariable fractional polynomial functions and logarithm hazard ratios for overall survival using variables in SEER.***

| Variables | Function | logHR | *P* value |
| --- | --- | --- | --- |
| *Continuous variables* |  |  |  |
| Age1 | ${(age/100)}^{-0.5}$ | -17.05 | <0.001 |
| Age2 | ${(age/100)}^{-0.5}\times log(age/100)$ | 14.78 | <0.001 |
| Tumor size, mm | $log(size/10)$ | 0.6767 | <0.001 |
| Node-positive ratio | $((ratio+0.1)/0.1)^{0.5}$ | 0.5963 | <0.001 |
| *Categorical varia*bles |  |  |  |
| Chemotherapy |  |  |  |
| without | $-$ | $-$ | $-$ |
| with | $-$ | -0.5638 | <0.001 |
| Radiotherapy |  |  |  |
| without | $-$ | $-$ | $-$ |
| with | $-$ | -0.3345 | <0.001 |
| Grade |  |  |  |
| 1 | $-$ | $-$ | $-$ |
| 2 | $-$ | 0.3070 | 0.025 |
| 3 | $-$ | 0.6743 | <0.001 |
| Subtype |  |  |  |
| Luminal-like | $-$ | $-$ | $-$ |
| HER2 | $-$ | 0.2491 | 0.0016 |
| Triple negative | $-$ | 1.19 | <0.001 |
| Pathological stage |  |  |  |
| 1 | $-$ | $-$ | $-$ |
| 2 | $-$ | 0.3355 | 0.0033 |
| 3 | $-$ | 0.8238 | <0.001 |
| 4 | $-$ | 1.721 | <0.001 |

*Derived from Model 3.

**Table S2. Multivariable fractional polynomial functions and logarithm hazard ratios for overall survival regressed on all variables.***

| Variables | Function | logHR | *P* value |
| --- | --- | --- | --- |
| *Continuous variables* |  |  |  |
| Age1 | ${(age/100)}^{-0.5}$ | -14.68 | 0.0035 |
| Age2 | ${(age/100)}^{-0.5}\times log(age/100)$ | 13.08 | <0.001 |
| Tumor size, mm | $log(size/10)$ | 0.589 | <0.001 |
| Node-positive ratio | $((ratio+0.1)/0.1)^{0.5}$ | 0.4337 | <0.001 |
| *Categorical variables* |  |  |  |
| Chemotherapy |  |  |  |
| without | $-$ | $-$ | $-$ |
| with | $-$ | -0.5011 | <0.001 |
| Radiotherapy |  |  |  |
| without | $-$ | $-$ | $-$ |
| with | $-$ | -0.3733 | <0.001 |
| Grade |  |  |  |
| 1 | $-$ | $-$ | $-$ |
| 2 | $-$ | 0.1327 | 0.317 |
| 3 | $-$ | 0.4415 | 0.001 |
| Subtype |  |  |  |
| Luminal-like | $-$ | $-$ | $-$ |
| HER2 | $-$ | 0.126 | 0.312 |
| Triple negative | $-$ | 0.6287 | <0.001 |
| Pathological stage |  |  |  |
| 1 | $-$ | $-$ | $-$ |
| 2 | $-$ | 0.2873 | 0.0126 |
| 3 | $-$ | 0.9391 | <0.001 |
| 4 | $-$ | 1.871 | <0.001 |
| Targeted therapy |  |  |  |
| without | $-$ | $-$ | $-$ |
| with | $-$ | -0.3533 | 0.0056 |
| Hormone/steroid therapy | | | |
| without | $-$ | $-$ | $-$ |
| with | $-$ | -0.5948 | <0.001 |
| Lymph vessel or vascular invasion | | | |
| No | $-$ | $-$ | $-$ |
| Yes | $-$ | 0.3005 | <0.001 |

*Derived from Model 4.

**Table S3. Model calibration regressed on all variables.**

|  | Calibration year | Observed | Predicted | *P* value |
| --- | --- | --- | --- | --- |
| Training data  (breast cancer-specific mortality) | 1 | 60 | 61.63 | 0.835 |
|  | 2 | 228 | 232.41 | 0.772 |
|  | 3 | 350 | 372.75 | 0.239 |
|  | 4 | 407 | 418.63 | 0.570 |
|  | 5 | 329 | 342.48 | 0.466 |
|  | 6 | 192 | 208.06 | 0.266 |
| Testing data  (breast cancer-specific mortality) | 1 | 24 | 31.57 | 0.178 |
|  | 2 | 107 | 118.58 | 0.288 |
|  | 3 | 186 | 189.80 | 0.783 |
|  | 4 | 213 | 208.98 | 0.781 |
|  | 5 | 187 | 169.40 | 0.176 |
|  | 6 | 117 | 104.23 | 0.211 |
| Training data  (overall mortality) | 1 | 88 | 89.26 | 0.894 |
|  | 2 | 304 | 305.08 | 0.951 |
|  | 3 | 470 | 473.97 | 0.855 |
|  | 4 | 549 | 539.59 | 0.685 |
|  | 5 | 445 | 444.25 | 0.972 |
|  | 6 | 261 | 260.13 | 0.957 |
| Testing data  (overall mortality) | 1 | 40 | 44.56 | 0.495 |
|  | 2 | 147 | 153.00 | 0.628 |
|  | 3 | 242 | 239.77 | 0.886 |
|  | 4 | 277 | 269.11 | 0.631 |
|  | 5 | 240 | 222.39 | 0.238 |
|  | 6 | 150 | 133.57 | 0.155 |

**Table S4.** **Model 1 calibration at 5 years in training data.**

|  | **Number of cases** | **Observed events** | **Predicted events** | **difference (%)** | ***P* value** |
| --- | --- | --- | --- | --- | --- |
| **Subtype** |  |  |  |  |  |
| Luminal-like | 3151 | 128 | 130.22 | -0.02 | 0.846 |
| HER2 | 1470 | 130 | 115.35 | 0.11 | 0.173 |
| Triple negative | 653 | 90 | 104.47 | -0.16 | 0.157 |
| **Grade** |  |  |  |  |  |
| 1 | 751 | 7 | 10.65 | -0.52 | 0.264 |
| 2 | 2660 | 145 | 136.47 | 0.06 | 0.465 |
| 3 | 1863 | 196 | 202.92 | -0.04 | 0.627 |
| **Pathological stage** |  |  |  |  |  |
| 1 | 2167 | 33 | 26.55 | 0.20 | 0.211 |
| 2 | 2116 | 97 | 113.04 | -0.17 | 0.131 |
| 3 | 858 | 154 | 152.23 | 0.01 | 0.886 |
| 4 | 133 | 64 | 58.22 | 0.09 | 0.449 |
| **Chemotherapy** |  |  |  |  |  |
| without | 1602 | 65 | 67.45 | -0.04 | 0.765 |
| with | 3672 | 283 | 282.59 | 0.00 | 0.981 |
| **Radiotherapy** |  |  |  |  |  |
| without | 2340 | 158 | 155.50 | 0.02 | 0.841 |
| with | 2934 | 190 | 194.54 | -0.02 | 0.745 |
| **Age at diagnosis** |  |  |  |  |  |
| <35 | 227 | 15 | 18.44 | -0.23 | 0.423 |
| 35 to 49 | 1867 | 110 | 103.14 | 0.06 | 0.499 |
| 50 to 64 | 2334 | 137 | 139.02 | -0.01 | 0.864 |
| 65 to 74 | 623 | 56 | 53.19 | 0.05 | 0.700 |
| 75+ | 223 | 30 | 36.26 | -0.21 | 0.298 |
| **Tumor size** |  |  |  |  |  |
| <10 | 669 | 6 | 4.40 | 0.27 | 0.445 |
| 10 to 19 | 1814 | 40 | 37.25 | 0.069 | 0.652 |
| 20 to 29 | 1385 | 80 | 83.98 | -05 | 0.664 |
| 30 to 49 | 971 | 125 | 119.86 | 0.041 | 0.639 |
| 50+ | 435 | 97 | 104.56 | -0.078 | 0.46 |

**Table S5. Model 1 calibration at 5 years in testing data.**

|  | **Number of cases** | **Observed events** | **Predicted events** | **difference (%)** | ***P* value** |
| --- | --- | --- | --- | --- | --- |
| **Subtype** |  |  |  |  |  |
| Luminal-like | 1660 | 68 | 63.77 | 0.06 | 0.597 |
| HER2 | 731 | 65 | 55.24 | 0.15 | 0.189 |
| Triple negative | 321 | 43 | 53.27 | -0.24 | 0.159 |
| **Grade** |  |  |  |  |  |
| 1 | 435 | 6 | 6.15 | -0.02 | 0.953 |
| 2 | 1341 | 70 | 59.52 | 0.15 | 0.174 |
| 3 | 936 | 100 | 106.62 | -0.07 | 0.522 |
| **Pathological stage** |  |  |  |  |  |
| 1 | 1095 | 18 | 13.37 | 0.26 | 0.206 |
| 2 | 1114 | 67 | 58.08 | 0.13 | 0.242 |
| 3 | 448 | 68 | 76.47 | -0.12 | 0.333 |
| 4 | 55 | 23 | 24.36 | -0.06 | 0.783 |
| **Chemotherapy** |  |  |  |  |  |
| without | 824 | 33 | 31.98 | 0.03 | 0.857 |
| with | 1888 | 143 | 140.30 | 0.02 | 0.820 |
| **Radiotherapy** |  |  |  |  |  |
| without | 1185 | 96 | 79.54 | 0.17 | 0.065 |
| with | 1527 | 80 | 92.74 | -0.16 | 0.186 |
| **Age at diagnosis** |  |  |  |  |  |
| <35 | 104 | 7 | 7.06 | -0.01 | 0.981 |
| 35 to 49 | 998 | 51 | 52.48 | -0.03 | 0.838 |
| 50 to 64 | 1181 | 66 | 67.79 | -0.03 | 0.828 |
| 65 to 74 | 304 | 39 | 26.44 | 0.32 | 0.015 |
| 75+ | 125 | 13 | 18.51 | -0.42 | 0.201 |
| **Tumor size** |  |  |  |  |  |
| <10 | 342 | 5 | 2.22 | 0.56 | 0.062 |
| 10 to 19 | 928 | 19 | 19.24 | -0.13 | 0.955 |
| 20 to 29 | 724 | 51 | 42.6 | 0.165 | 0.198 |
| 30 to 49 | 519 | 63 | 59.48 | 0.056 | 0.648 |
| 50+ | 199 | 38 | 48.74 | -0.283 | 0.124 |

**Table S6. Model 1 calibration at 3 years in training data.**

|  | **Number of cases** | **Observed events** | **Predicted events** | **difference (%)** | ***P* value** |
| --- | --- | --- | --- | --- | --- |
| **Subtype** |  |  |  |  |  |
| Luminal-like | 6947 | 126 | 143.85 | -0.14 | 0.137 |
| HER2 | 2930 | 107 | 123.23 | -0.15 | 0.144 |
| Triple negative | 1212 | 130 | 106.37 | 0.18 | 0.022 |
| **Grade** |  |  |  |  |  |
| 1 | 1786 | 8 | 12.40 | -0.55 | 0.21 |
| 2 | 5605 | 131 | 143.16 | -0.09 | 0.31 |
| 3 | 3698 | 224 | 217.89 | 0.03 | 0.68 |
| **Pathological stage** |  |  |  |  |  |
| 1 | 4564 | 21 | 26.18 | -0.25 | 0.312 |
| 2 | 4546 | 115 | 113.37 | 0.01 | 0.878 |
| 3 | 1713 | 162 | 162.89 | -0.01 | 0.944 |
| 4 | 266 | 65 | 71.02 | -0.09 | 0.475 |
| **Chemotherapy** |  |  |  |  |  |
| without | 3678 | 60 | 75.69 | -0.26 | 0.071 |
| with | 7411 | 303 | 297.76 | 0.02 | 0.761 |
| **Radiotherapy** |  |  |  |  |  |
| without | 4944 | 155 | 173.71 | -0.12 | 0.156 |
| with | 6145 | 208 | 199.74 | 0.04 | 0.559 |
| **Age at diagnosis** |  |  |  |  |  |
| <35 | 440 | 19 | 18.64 | 0.02 | 0.933 |
| 35 to 49 | 3913 | 105 | 106.06 | -0.01 | 0.918 |
| 50 to 64 | 4885 | 150 | 151.27 | -0.01 | 0.918 |
| 65 to 74 | 1354 | 56 | 58.80 | -0.05 | 0.715 |
| 75+ | 497 | 33 | 38.68 | -0.17 | 0.361 |
| **Tumor size** |  |  |  |  |  |
| <10 | 1429 | 4 | 4.26 | -0.06 | 0.901 |
| 10 to 19 | 3787 | 34 | 37.3 | -0.1 | 0.589 |
| 20 to 29 | 2957 | 74 | 85.74 | -0.16 | 0.205 |
| 30 to 49 | 2040 | 129 | 121.38 | 0.06 | 0.489 |
| 50+ | 876 | 122 | 124.78 | -0.02 | 0.804 |

**Table S7.** **Model 1 calibration at 3 years in testing data.**

|  | **Number of cases** | **Observed events** | **Predicted events** | **difference (%)** | ***P* value** |
| --- | --- | --- | --- | --- | --- |
| **Subtype** |  |  |  |  |  |
| Luminal-like | 3577 | 70 | 70.53 | -0.01 | 0.950 |
| HER2 | 1497 | 55 | 59.53 | -0.08 | 0.558 |
| Triple negative | 576 | 52 | 52.75 | -0.01 | 0.917 |
| **Grade** |  |  |  |  |  |
| 1 | 924 | 3 | 6.14 | -1.05 | 0.205 |
| 2 | 2846 | 56 | 64.43 | -0.15 | 0.294 |
| 3 | 1880 | 118 | 112.24 | 0.05 | 0.587 |
| **Pathological stage** |  |  |  |  |  |
| 1 | 2333 | 14 | 13.74 | 0.02 | 0.944 |
| 2 | 2332 | 59 | 60.04 | -0.02 | 0.893 |
| 3 | 860 | 73 | 77.36 | -0.06 | 0.620 |
| 4 | 125 | 31 | 31.67 | -0.02 | 0.905 |
| **Chemotherapy** |  |  |  |  |  |
| without | 1847 | 39 | 39.12 | 0.00 | 0.985 |
| with | 3803 | 138 | 143.69 | -0.04 | 0.635 |
| **Radiotherapy** |  |  |  |  |  |
| without | 2504 | 99 | 89.25 | 0.10 | 0.302 |
| with | 3146 | 78 | 93.56 | -0.20 | 0.108 |
| **Age at diagnosis** |  |  |  |  |  |
| <35 | 209 | 6 | 6.69 | -0.12 | 0.789 |
| 35 to 49 | 1993 | 44 | 50.95 | -0.16 | 0.330 |
| 50 to 64 | 2494 | 74 | 73.21 | 0.01 | 0.926 |
| 65 to 74 | 673 | 32 | 29.97 | 0.06 | 0.711 |
| 75+ | 281 | 21 | 21.98 | -0.05 | 0.834 |
| **Tumor size** |  |  |  |  |  |
| <10 | 694 | 3 | 2.09 | 0.30 | 0.528 |
| 10 to 19 | 1966 | 19 | 20.01 | -0.05 | 0.822 |
| 20 to 29 | 1484 | 39 | 41.74 | -0.07 | 0.672 |
| 30 to 49 | 1071 | 65 | 59.28 | 0.09 | 0.458 |
| 50+ | 435 | 51 | 59.69 | -0.17 | 0.261 |

**Table S8.** **Model 3 calibration at 5 years in training data.**

|  | **Number of cases** | **Observed events** | **Predicted events** | **difference (%)** | ***P* value** |
| --- | --- | --- | --- | --- | --- |
| **Subtype** |  |  |  |  |  |
| Luminal-like | 3151 | 199 | 192.80 | 0.03 | 0.655 |
| HER2 | 1470 | 155 | 143.45 | 0.07 | 0.335 |
| Triple negative | 653 | 112 | 120.05 | -0.07 | 0.462 |
| **Grade** |  |  |  |  |  |
| 1 | 751 | 19 | 24.32 | -0.28 | 0.281 |
| 2 | 2660 | 202 | 189.26 | 0.06 | 0.354 |
| 3 | 1863 | 245 | 242.72 | 0.01 | 0.884 |
| **Pathological stage** |  |  |  |  |  |
| 1 | 2167 | 65 | 57.41 | 0.12 | 0.316 |
| 2 | 2116 | 144 | 159.05 | -0.10 | 0.233 |
| 3 | 858 | 186 | 178.07 | 0.04 | 0.553 |
| 4 | 133 | 71 | 61.77 | 0.13 | 0.240 |
| **Chemotherapy** |  |  |  |  |  |
| without | 1602 | 120 | 119.61 | 0.003 | 0.972 |
| with | 3672 | 346 | 336.69 | 0.03 | 0.612 |
| **Radiotherapy** |  |  |  |  |  |
| without | 2340 | 234 | 221.84 | 0.05 | 0.414 |
| with | 2934 | 232 | 234.46 | -0.01 | 0.872 |
| **Age at diagnosis** |  |  |  |  |  |
| <35 | 227 | 16 | 19.53 | -0.22 | 0.424 |
| 35 to 49 | 1867 | 122 | 115.90 | 0.05 | 0.571 |
| 50 to 64 | 2334 | 177 | 177.01 | -0.0001 | 0.999 |
| 65 to 74 | 623 | 89 | 81.37 | 0.09 | 0.398 |
| 75+ | 223 | 62 | 62.49 | -0.01 | 0.951 |
| **Tumor size** |  |  |  |  |  |
| <10 | 669 | 14 | 10.85 | 0.22 | 0.339 |
| 10 to 19 | 1814 | 69 | 66.84 | 0.03 | 0.792 |
| 20 to 29 | 1385 | 109 | 116.74 | -0.07 | 0.474 |
| 30 to 49 | 971 | 163 | 147.5 | 0.1 | 0.202 |
| 50+ | 435 | 111 | 114.37 | -0.03 | 0.753 |

**Table S9.** **Model 3 calibration at 5 years in testing data.**

|  | **Number of cases** | **Observed events** | **Predicted events** | **difference (%)** | ***P* value** |
| --- | --- | --- | --- | --- | --- |
| **Subtype** |  |  |  |  |  |
| Luminal-like | 1660 | 97 | 97.74 | -0.01 | 0.941 |
| HER2 | 731 | 76 | 68.27 | 0.10 | 0.349 |
| Triple negative | 321 | 54 | 60.75 | -0.12 | 0.387 |
| **Grade** |  |  |  |  |  |
| 1 | 435 | 12 | 14.16 | -0.18 | 0.566 |
| 2 | 1341 | 92 | 86.48 | 0.06 | 0.553 |
| 3 | 936 | 123 | 126.11 | -0.03 | 0.782 |
| **Pathological stage** |  |  |  |  |  |
| 1 | 1095 | 36 | 29.19 | 0.19 | 0.208 |
| 2 | 1114 | 87 | 82.02 | 0.06 | 0.582 |
| 3 | 448 | 79 | 89.25 | -0.13 | 0.278 |
| 4 | 55 | 25 | 26.29 | -0.05 | 0.801 |
| **Chemotherapy** |  |  |  |  |  |
| without | 824 | 58 | 58.38 | -0.01 | 0.960 |
| with | 1888 | 169 | 168.37 | 0.004 | 0.961 |
| **Radiotherapy** |  |  |  |  |  |
| without | 1185 | 128 | 113.42 | 0.11 | 0.171 |
| with | 1527 | 99 | 113.33 | -0.14 | 0.178 |
| **Age at diagnosis** |  |  |  |  |  |
| <35 | 104 | 8 | 7.86 | 0.02 | 0.959 |
| 35 to 49 | 998 | 61 | 59.13 | 0.03 | 0.808 |
| 50 to 64 | 1181 | 75 | 87.07 | -0.16 | 0.196 |
| 65 to 74 | 304 | 56 | 40.88 | 0.27 | 0.018 |
| 75+ | 125 | 27 | 31.81 | -0.18 | 0.394 |
| **Tumor size** |  |  |  |  |  |
| <10 | 342 | 7 | 5.35 | 0.24 | 0.477 |
| 10 to 19 | 928 | 40 | 35.29 | 0.12 | 0.427 |
| 20 to 29 | 724 | 62 | 58.5 | 0.06 | 0.646 |
| 30 to 49 | 519 | 77 | 75.05 | 0.02 | 0.822 |
| 50+ | 199 | 41 | 52.58 | -0.28 | 0.110 |

**Table S10.** **Model 3 calibration at 3 years in training data.**

|  | **Number of cases** | **Observed events** | **Predicted events** | **difference (%)** | ***P* value** |
| --- | --- | --- | --- | --- | --- |
| **Subtype** |  |  |  |  |  |
| Luminal-like | 6947 | 194 | 209.40 | -0.08 | 0.287 |
| HER2 | 2930 | 141 | 148.57 | -0.05 | 0.535 |
| Triple negative | 1212 | 146 | 117.74 | 0.19 | 0.010 |
| **Grade** |  |  |  |  |  |
| 1 | 1786 | 19 | 27.88 | -0.47 | 0.093 |
| 2 | 5605 | 192 | 197.14 | -0.03 | 0.715 |
| 3 | 3698 | 270 | 250.69 | 0.07 | 0.223 |
| **Pathological stage** |  |  |  |  |  |
| 1 | 4564 | 53 | 56.33 | -0.06 | 0.658 |
| 2 | 4546 | 160 | 158.66 | 0.01 | 0.916 |
| 3 | 1713 | 192 | 185.64 | 0.03 | 0.641 |
| 4 | 266 | 76 | 75.08 | 0.01 | 0.915 |
| **Chemotherapy** |  |  |  |  |  |
| without | 3678 | 117 | 133.32 | -0.14 | 0.157 |
| with | 7411 | 364 | 342.38 | 0.06 | 0.243 |
| **Radiotherapy** |  |  |  |  |  |
| without | 4944 | 240 | 242.16 | -0.01 | 0.889 |
| with | 6145 | 241 | 233.54 | 0.03 | 0.626 |
| **Age at diagnosis** |  |  |  |  |  |
| <35 | 440 | 20 | 18.85 | 0.06 | 0.791 |
| 35 to 49 | 3913 | 116 | 113.68 | 0.02 | 0.828 |
| 50 to 64 | 4885 | 195 | 184.79 | 0.05 | 0.452 |
| 65 to 74 | 1354 | 83 | 89.20 | -0.07 | 0.511 |
| 75+ | 497 | 67 | 69.19 | -0.03 | 0.793 |
| **Tumor size** |  |  |  |  |  |
| <10 | 1429 | 13 | 10.59 | 0.19 | 0.460 |
| 10 to 19 | 3787 | 60 | 66.23 | -0.10 | 0.444 |
| 20 to 29 | 2957 | 108 | 118.06 | -0.09 | 0.355 |
| 30 to 49 | 2040 | 157 | 148.58 | 0.05 | 0.49 |
| 50+ | 876 | 143 | 132.24 | 0.08 | 0.35 |

**Table S11.** **Model 3 calibration at 3 years in testing data.**

|  | **Number of cases** | **Observed events** | **Predicted events** | **difference (%)** | ***P* value** |
| --- | --- | --- | --- | --- | --- |
| **Subtype** |  |  |  |  |  |
| Luminal-like | 3577 | 109 | 105.10 | 0.04 | 0.704 |
| HER2 | 1497 | 66 | 73.08 | -0.11 | 0.407 |
| Triple negative | 576 | 62 | 59.29 | 0.04 | 0.725 |
| **Grade** |  |  |  |  |  |
| 1 | 924 | 14 | 14.04 | -0.003 | 0.991 |
| 2 | 2846 | 80 | 92.35 | -0.15 | 0.199 |
| 3 | 1880 | 143 | 131.08 | 0.08 | 0.298 |
| **Pathological stage** |  |  |  |  |  |
| 1 | 2333 | 34 | 29.15 | 0.14 | 0.369 |
| 2 | 2332 | 85 | 84.19 | 0.01 | 0.930 |
| 3 | 860 | 84 | 91.01 | -0.08 | 0.462 |
| 4 | 125 | 34 | 33.12 | 0.03 | 0.879 |
| **Chemotherapy** |  |  |  |  |  |
| without | 1847 | 72 | 69.57 | 0.03 | 0.771 |
| with | 3803 | 165 | 167.90 | -0.02 | 0.823 |
| **Radiotherapy** |  |  |  |  |  |
| without | 2504 | 140 | 125.03 | 0.11 | 0.181 |
| with | 3146 | 97 | 112.45 | -0.16 | 0.145 |
| **Age at diagnosis** |  |  |  |  |  |
| <35 | 209 | 6 | 7.14 | -0.19 | 0.669 |
| 35 to 49 | 1993 | 53 | 55.21 | -0.04 | 0.767 |
| 50 to 64 | 2494 | 87 | 90.48 | -0.04 | 0.714 |
| 65 to 74 | 673 | 49 | 45.36 | 0.07 | 0.588 |
| 75+ | 281 | 42 | 39.29 | 0.06 | 0.665 |
| **Tumor size** |  |  |  |  |  |
| <10 | 694 | 8 | 5.09 | 0.36 | 0.197 |
| 10 to 19 | 1966 | 36 | 35.51 | 0.01 | 0.934 |
| 20 to 29 | 1484 | 55 | 57.54 | -0.05 | 0.738 |
| 30 to 49 | 1071 | 81 | 75.89 | 0.06 | 0.557 |
| 50+ | 435 | 57 | 63.45 | -0.11 | 0.418 |

**Table S12.** **Model 3 calibration at 3 years in the Asian population of the SEER database.**

|  | **Number of cases** | **Observed events** | **Predicted events** | **difference (%)** | ***P* value** |
| --- | --- | --- | --- | --- | --- |
| **Subtype** |  |  |  |  |  |
| Luminal-like | 854 | 28 | 24.78 | 0.12 | 0.518 |
| HER2 | 181 | 3 | 7.25 | -1.42 | 0.115 |
| Triple negative | 110 | 12 | 12.13 | -0.01 | 0.970 |
| **Grade** |  |  |  |  |  |
| 1 | 261 | 2 | 4.17 | -1.09 | 0.287 |
| 2 | 538 | 13 | 18.48 | -0.42 | 0.202 |
| 3 | 346 | 28 | 21.50 | 0.23 | 0.161 |
| **Pathological stage** |  |  |  |  |  |
| 1 | 577 | 7 | 8.41 | -0.20 | 0.626 |
| 2 | 457 | 18 | 21.21 | -0.18 | 0.486 |
| 3 | 102 | 15 | 11.52 | 0.23 | 0.306 |
| 4 | 9 | 3 | 3.01 | 0.00 | 0.996 |
| **Chemotherapy** |  |  |  |  |  |
| without | 630 | 18 | 20.18 | -0.12 | 0.627 |
| with | 515 | 25 | 23.97 | 0.04 | 0.834 |
| **Radiotherapy** |  |  |  |  |  |
| without | 397 | 24 | 18.13 | 0.24 | 0.168 |
| with | 748 | 19 | 26.03 | -0.37 | 0.168 |
| **Age at diagnosis** |  |  |  |  |  |
| <35 | 17 | 2 | 0.58 | 0.71 | 0.060 |
| 35 to 49 | 333 | 7 | 8.31 | -0.19 | 0.650 |
| 50 to 64 | 428 | 11 | 13.93 | -0.27 | 0.432 |
| 65 to 74 | 206 | 8 | 8.99 | -0.12 | 0.741 |
| 75+ | 161 | 15 | 12.35 | 0.18 | 0.451 |
| **Tumor size** |  |  |  |  |  |
| <10 | 260 | 3 | 2.61 | 0.13 | 0.811 |
| 10 to 19 | 388 | 6 | 9.12 | -0.52 | 0.301 |
| 20 to 29 | 257 | 9 | 10.57 | -0.17 | 0.629 |
| 30 to 49 | 161 | 18 | 11.73 | 0.35 | 0.067 |
| 50+ | 79 | 7 | 10.12 | -0.45 | 0.326 |

**Table S13. Model 2 calibration at 5 years in training data.**

|  | **Number of cases** | **Observed events** | **Predicted events** | **difference (%)** | ***P* value** |
| --- | --- | --- | --- | --- | --- |
| **Subtype** |  |  |  |  |  |
| Luminal-like | 3128 | 121 | 131.94 | -0.09 | 0.341 |
| HER2 | 1466 | 135 | 118.21 | 0.12 | 0.123 |
| Triple negative | 602 | 73 | 92.32 | -0.26 | 0.044 |
| **Grade** |  |  |  |  |  |
| 1 | 749 | 8 | 11.44 | -0.43 | 0.309 |
| 2 | 2607 | 139 | 132.51 | 0.05 | 0.573 |
| 3 | 1840 | 182 | 198.53 | -0.09 | 0.241 |
| **Pathological stage** |  |  |  |  |  |
| 1 | 2082 | 31 | 27.36 | 0.12 | 0.487 |
| 2 | 2107 | 90 | 106.79 | -0.19 | 0.104 |
| 3 | 881 | 153 | 152.82 | 0.001 | 0.988 |
| 4 | 126 | 55 | 55.51 | -0.01 | 0.945 |
| **Chemotherapy** |  |  |  |  |  |
| without | 1558 | 58 | 61.84 | -0.07 | 0.625 |
| with | 3638 | 271 | 280.64 | -0.04 | 0.565 |
| **Radiotherapy** |  |  |  |  |  |
| without | 2311 | 154 | 159.72 | -0.04 | 0.651 |
| with | 2885 | 175 | 182.76 | -0.04 | 0.566 |
| **Age at diagnosis** |  |  |  |  |  |
| <35 | 212 | 11 | 12.96 | -0.18 | 0.586 |
| 35 to 49 | 1864 | 107 | 98.78 | 0.08 | 0.408 |
| 50 to 64 | 2312 | 131 | 146.87 | -0.12 | 0.190 |
| 65 to 74 | 590 | 58 | 50.45 | 0.13 | 0.288 |
| 75+ | 218 | 22 | 33.43 | -0.52 | 0.048 |
| **Tumor size** |  |  |  |  |  |
| <10 | 669 | 7 | 5 | 0.29 | 0.371 |
| 10 to 19 | 1726 | 38 | 36.89 | 0.03 | 0.845 |
| 20 to 29 | 1396 | 83 | 84 | -0.01 | 0.913 |
| 30 to 49 | 974 | 115 | 115.59 | -0.005 | 0.956 |
| 50+ | 431 | 86 | 101 | -0.17 | 0.135 |
| **Hormone/steroid therapy** | | | | | |
| without | 1581 | 198 | 204.76 | -0.03 | 0.637 |
| with | 3615 | 131 | 137.72 | -0.05 | 0.567 |
| **Targeted therapy** |  |  |  |  |  |
| without | 4303 | 236 | 259.24 | -0.10 | 0.149 |
| with | 893 | 93 | 83.24 | 0.10 | 0.285 |
| **Lymph vessels or vascular invasion** | | | | | |
| No | 3213 | 105 | 110.78 | -0.06 | 0.583 |
| Yes | 1983 | 224 | 231.70 | -0.03 | 0.613 |

**Table S14. Model 2 calibration at 5 years in testing data.**

|  | **Number of cases** | **Observed events** | **Predicted events** | **difference (%)** | ***P* value** |
| --- | --- | --- | --- | --- | --- |
| **Subtype** |  |  |  |  |  |
| Luminal-like | 1588 | 74 | 64.15 | 0.13 | 0.219 |
| HER2 | 679 | 54 | 48.65 | 0.10 | 0.443 |
| Triple negative | 351 | 59 | 56.60 | 0.04 | 0.749 |
| **Grade** |  |  |  |  |  |
| 1 | 409 | 5 | 6.67 | -0.33 | 0.518 |
| 2 | 1300 | 73 | 61.76 | 0.15 | 0.153 |
| 3 | 909 | 109 | 100.98 | 0.07 | 0.425 |
| **Pathological stage** |  |  |  |  |  |
| 1 | 1090 | 19 | 14.04 | 0.26 | 0.186 |
| 2 | 1064 | 70 | 54.51 | 0.22 | 0.036 |
| 3 | 405 | 67 | 73.76 | -0.10 | 0.431 |
| 4 | 59 | 31 | 27.09 | 0.13 | 0.453 |
| **Chemotherapy** |  |  |  |  |  |
| without | 804 | 39 | 37.42 | 0.04 | 0.796 |
| with | 1814 | 148 | 131.98 | 0.11 | 0.163 |
| **Radiotherapy** |  |  |  |  |  |
| without | 1151 | 95 | 77.43 | 0.18 | 0.046 |
| with | 1467 | 92 | 91.97 | 0.0003 | 0.998 |
| **Age at diagnosis** |  |  |  |  |  |
| <35 | 104 | 11 | 9.46 | 0.14 | 0.616 |
| 35 to 49 | 933 | 49 | 48.94 | 0.001 | 0.993 |
| 50 to 64 | 1132 | 69 | 62.10 | 0.10 | 0.381 |
| 65 to 74 | 321 | 37 | 27.65 | 0.25 | 0.075 |
| 75+ | 128 | 21 | 21.26 | -0.01 | 0.956 |
| **Tumor size** |  |  |  |  |  |
| <10 | 311 | 4 | 2.25 | 0.44 | 0.243 |
| 10 to 19 | 947 | 20 | 19.99 | 0.0005 | 0.998 |
| 20 to 29 | 674 | 45 | 40.09 | 0.11 | 0.438 |
| 30 to 49 | 493 | 70 | 60.56 | 0.14 | 0.225 |
| 50+ | 193 | 48 | 46.51 | 0.03 | 0.827 |
| **Hormone/steroid therapy** | | | | | |
| without | 798 | 102 | 102.62 | -0.01 | 0.951 |
| with | 1820 | 85 | 66.78 | 0.21 | 0.026 |
| **Targeted therapy** |  |  |  |  |  |
| without | 2209 | 150 | 136.71 | 0.09 | 0.256 |
| with | 409 | 37 | 32.69 | 0.12 | 0.451 |
| **Lymph vessels or vascular invasion** | | | | | |
| No | 1680 | 71 | 56.85 | 0.20 | 0.061 |
| Yes | 938 | 116 | 112.55 | 0.03 | 0.745 |

**Table S15. Model 2 calibration at 3 years in training data.**

|  | **Number of cases** | **Observed events** | **Predicted events** | **difference (%)** | ***P* value** |
| --- | --- | --- | --- | --- | --- |
| **Subtype** |  |  |  |  |  |
| Luminal-like | 6884 | 130 | 151.19 | -0.16 | 0.085 |
| HER2 | 2915 | 109 | 123.10 | -0.13 | 0.204 |
| Triple negative | 1131 | 111 | 98.46 | 0.11 | 0.206 |
| **Grade** |  |  |  |  |  |
| 1 | 1744 | 7 | 13.29 | -0.90 | 0.084 |
| 2 | 5507 | 124 | 143.02 | -0.15 | 0.112 |
| 3 | 3679 | 219 | 216.44 | 0.01 | 0.862 |
| **Pathological stage** |  |  |  |  |  |
| 1 | 4481 | 22 | 28.77 | -0.31 | 0.207 |
| 2 | 4488 | 106 | 111.32 | -0.05 | 0.614 |
| 3 | 1713 | 161 | 166.78 | -0.04 | 0.654 |
| 4 | 248 | 61 | 65.88 | -0.08 | 0.548 |
| **Chemotherapy** |  |  |  |  |  |
| without | 3590 | 69 | 78.78 | -0.14 | 0.271 |
| with | 7340 | 281 | 293.97 | -0.05 | 0.449 |
| **Radiotherapy** |  |  |  |  |  |
| without | 4890 | 166 | 183.47 | -0.11 | 0.197 |
| with | 6040 | 184 | 189.28 | -0.03 | 0.701 |
| **Age at diagnosis** |  |  |  |  |  |
| <35 | 425 | 12 | 13.92 | -0.16 | 0.608 |
| 35 to 49 | 3846 | 95 | 100.12 | -0.05 | 0.609 |
| 50 to 64 | 4861 | 151 | 158.79 | -0.05 | 0.536 |
| 65 to 74 | 1298 | 56 | 58.58 | -0.05 | 0.736 |
| 75+ | 500 | 36 | 41.34 | -0.15 | 0.406 |
| **Tumor size** |  |  |  |  |  |
| <10 | 1406 | 5 | 5.00 | 0.0007 | 0.999 |
| 10 to 19 | 3692 | 33 | 38.96 | -0.18 | 0.34 |
| 20 to 29 | 2897 | 72 | 84.32 | -0.17 | 0.18 |
| 30 to 49 | 2072 | 124 | 122.59 | 0.01 | 0.898 |
| 50+ | 863 | 116 | 121.88 | -0.05 | 0.594 |
| **Hormone/steroid therapy** | | | | | |
| without | 2987 | 222 | 216.05 | 0.03 | 0.685 |
| with | 7943 | 128 | 156.70 | -0.22 | 0.022 |
| **Targeted therapy** |  |  |  |  |  |
| without | 9101 | 267 | 284.93 | -0.07 | 0.288 |
| with | 1829 | 83 | 87.82 | -0.06 | 0.607 |
| **Lymph vessels or vascular invasion** | | | | | |
| No | 6894 | 110 | 117.35 | -0.07 | 0.497 |
| Yes | 4036 | 240 | 255.39 | -0.06 | 0.335 |

**Table S16. Model 2 calibration at 3 years in testing data.**

|  | **Number of cases** | **Observed events** | **Predicted events** | **difference (%)** | ***P* value** | |
| --- | --- | --- | --- | --- | --- | --- |
| **Subtype** |  |  |  |  |  | |
| Luminal-like | 3451 | 66 | 75.88 | -0.15 | 0.257 | |
| HER2 | 1412 | 52 | 58.01 | -0.12 | 0.430 | |
| Triple negative | 618 | 68 | 55.91 | 0.18 | 0.106 | |
| **Grade** |  |  |  |  |  | |
| 1 | 909 | 4 | 7.03 | -0.76 | 0.253 | |
| 2 | 2771 | 62 | 72.07 | -0.16 | 0.235 | |
| 3 | 1801 | 120 | 110.69 | 0.08 | 0.376 | |
| **Pathological stage** |  |  |  |  |  | |
| 1 | 2258 | 13 | 14.15 | -0.09 | 0.760 | |
| 2 | 2260 | 66 | 57.93 | 0.12 | 0.289 | |
| 3 | 829 | 72 | 79.18 | -0.10 | 0.420 | |
| 4 | 134 | 35 | 38.54 | -0.10 | 0.568 | |
| **Chemotherapy** |  |  |  |  |  | |
| without | 1812 | 30 | 40.40 | -0.35 | 0.102 | |
| with | 3669 | 156 | 149.40 | 0.04 | 0.589 | |
| **Radiotherapy** |  |  |  |  |  | |
| without | 2429 | 85 | 90.09 | -0.06 | 0.592 | |
| with | 3052 | 101 | 99.70 | 0.01 | 0.897 | |
| **Age at diagnosis** |  |  |  |  |  | |
| <35 | 201 | 13 | 9.43 | 0.27 | 0.246 | |
| 35 to 49 | 1935 | 53 | 54.39 | -0.03 | 0.851 | |
| 50 to 64 | 2382 | 71 | 75.36 | -0.06 | 0.616 | |
| 65 to 74 | 693 | 32 | 30.03 | 0.06 | 0.719 | |
| 75+ | 270 | 17 | 20.59 | -0.21 | 0.429 | |
| **Tumor size** |  |  |  |  |  | |
| <10 | 657 | 2 | 2.31 | -0.16 | 0.837 | |
| 10 to 19 | 1945 | 20 | 21.49 | -0.07 | 0.748 | |
| 20 to 29 | 1467 | 40 | 45.6 | -0.14 | 0.407 | |
| 30 to 49 | 983 | 68 | 60 | 0.12 | 0.301 | |
| 50+ | 429 | 56 | 60.4 | -0.08 | 0.571 | |
| **Hormone/steroid therapy** | | | | | | |
| without | 1553 | 122 | 111.87 | 0.08 | | 0.338 |
| with | 3928 | 64 | 77.92 | -0.22 | | 0.115 |
| **Targeted therapy** |  |  |  |  | |  |
| without | 4595 | 145 | 149.52 | -0.03 | | 0.711 |
| with | 886 | 41 | 40.27 | 0.02 | | 0.909 |
| **Lymph vessels or vascular invasion** | | | | | | |
| No | 3485 | 58 | 60.19 | -0.04 | | 0.777 |
| Yes | 1996 | 128 | 129.60 | -0.01 | | 0.888 |

**Table S17. Model 4 calibration at 5 years in training data.**

|  | **Number of cases** | **Observed events** | **Predicted events** | **difference (%)** | ***P* value** |
| --- | --- | --- | --- | --- | --- |
| **Subtype** |  |  |  |  |  |
| Luminal-like | 3128 | 187 | 194.41 | -0.04 | 0.595 |
| HER2 | 1466 | 160 | 141.70 | 0.11 | 0.124 |
| Triple negative | 602 | 98 | 108.15 | -0.10 | 0.329 |
| **Grade** |  |  |  |  |  |
| 1 | 749 | 23 | 26.69 | -0.16 | 0.475 |
| 2 | 2607 | 192 | 181.12 | 0.06 | 0.419 |
| 3 | 1840 | 230 | 236.45 | -0.03 | 0.675 |
| **Pathological stage** |  |  |  |  |  |
| 1 | 2082 | 62 | 58.32 | 0.06 | 0.629 |
| 2 | 2107 | 133 | 149.78 | -0.13 | 0.170 |
| 3 | 881 | 188 | 178.09 | 0.05 | 0.458 |
| 4 | 126 | 62 | 58.07 | 0.06 | 0.606 |
| **Chemotherapy** |  |  |  |  |  |
| without | 1558 | 114 | 111.23 | 0.02 | 0.793 |
| with | 3638 | 331 | 333.02 | -0.01 | 0.912 |
| **Radiotherapy** |  |  |  |  |  |
| without | 2311 | 230 | 225.32 | 0.02 | 0.755 |
| with | 2885 | 215 | 218.93 | -0.02 | 0.791 |
| **Age at diagnosis** |  |  |  |  |  |
| <35 | 212 | 13 | 14.41 | -0.11 | 0.710 |
| 35 to 49 | 1864 | 124 | 112.31 | 0.09 | 0.270 |
| 50 to 64 | 2312 | 166 | 183.39 | -0.10 | 0.199 |
| 65 to 74 | 590 | 90 | 76.92 | 0.15 | 0.136 |
| 75+ | 218 | 52 | 57.22 | -0.10 | 0.490 |
| **Tumor size** |  |  |  |  |  |
| <10 | 669 | 15 | 12.31 | 0.18 | 0.444 |
| 10 to 19 | 1726 | 69 | 66.28 | 0.04 | 0.738 |
| 20 to 29 | 1396 | 115 | 115.86 | -0.007 | 0.936 |
| 30 to 49 | 974 | 147 | 141.06 | 0.04 | 0.618 |
| 50+ | 431 | 99 | 108.73 | -0.1 | 0.351 |
| **Hormone/steroid therapy** | | | | | |
| without | 1581 | 244 | 236.64 | 0.03 | 0.632 |
| with | 3615 | 201 | 207.62 | -0.03 | 0.646 |
| **Targeted therapy** |  |  |  |  |  |
| without | 4303 | 337 | 347.98 | -0.03 | 0.556 |
| with | 893 | 108 | 96.28 | 0.11 | 0.232 |
| **Lymph vessels or vascular invasion** | | | | | |
| No | 3213 | 167 | 167.13 | -0.001 | 0.992 |
| Yes | 1983 | 278 | 277.13 | 0.003 | 0.958 |

**Table S18. Model 4 calibration at 5 years in testing data.**

|  | **Number of cases** | **Observed events** | **Predicted events** | **difference (%)** | ***P* value** |
| --- | --- | --- | --- | --- | --- |
| **Subtype** |  |  |  |  |  |
| Luminal-like | 1588 | 108 | 97.12 | 0.10 | 0.270 |
| HER2 | 679 | 65 | 60.95 | 0.06 | 0.604 |
| Triple negative | 351 | 67 | 64.32 | 0.04 | 0.738 |
| **Grade** |  |  |  |  |  |
| 1 | 409 | 8 | 15.61 | -0.95 | 0.054 |
| 2 | 1300 | 99 | 85.75 | 0.13 | 0.153 |
| 3 | 909 | 133 | 121.03 | 0.09 | 0.277 |
| **Pathological stage** |  |  |  |  |  |
| 1 | 1090 | 38 | 29.92 | 0.21 | 0.140 |
| 2 | 1064 | 94 | 76.96 | 0.18 | 0.052 |
| 3 | 405 | 75 | 86.37 | -0.15 | 0.221 |
| 4 | 59 | 33 | 29.14 | 0.12 | 0.474 |
| **Chemotherapy** |  |  |  |  |  |
| without | 804 | 63 | 64.39 | -0.02 | 0.862 |
| with | 1814 | 177 | 158.00 | 0.11 | 0.131 |
| **Radiotherapy** |  |  |  |  |  |
| without | 1151 | 127 | 112.14 | 0.12 | 0.161 |
| with | 1467 | 113 | 110.25 | 0.02 | 0.793 |
| **Age at diagnosis** |  |  |  |  |  |
| <35 | 104 | 11 | 9.79 | 0.11 | 0.698 |
| 35 to 49 | 933 | 54 | 55.58 | -0.03 | 0.832 |
| 50 to 64 | 1132 | 83 | 80.24 | 0.03 | 0.758 |
| 65 to 74 | 321 | 55 | 41.45 | 0.25 | 0.035 |
| 75+ | 128 | 37 | 35.34 | 0.04 | 0.779 |
| **Tumor size** |  |  |  |  |  |
| <10 | 311 | 6 | 5.48 | 0.09 | 0.823 |
| 10 to 19 | 947 | 39 | 35.8 | 0.08 | 0.593 |
| 20 to 29 | 674 | 53 | 55.47 | -0.05 | 0.74 |
| 30 to 49 | 493 | 90 | 75.43 | 0.16 | 0.093 |
| 50+ | 193 | 52 | 50.2 | 0.03 | 0.8 |
| **Hormone/steroid therapy** | | | | | |
| without | 798 | 120 | 118.44 | 0.01 | 0.886 |
| with | 1820 | 120 | 103.94 | 0.13 | 0.115 |
| **Targeted therapy** |  |  |  |  |  |
| without | 2209 | 199 | 182.66 | 0.08 | 0.227 |
| with | 409 | 41 | 39.73 | 0.03 | 0.840 |
| **Lymph vessels or vascular invasion** | | | | | |
| No | 1680 | 101 | 85.86 | 0.15 | 0.102 |
| Yes | 938 | 139 | 136.53 | 0.02 | 0.833 |

**Table S19. Model 4 calibration at 3 years in training data.**

|  | **Number of cases** | **Observed events** | **Predicted events** | **difference (%)** | ***P* value** |
| --- | --- | --- | --- | --- | --- |
| **Subtype** |  |  |  |  |  |
| Luminal-like | 6884 | 201 | 217.65 | -0.08 | 0.259 |
| HER2 | 2915 | 140 | 145.24 | -0.04 | 0.664 |
| Triple negative | 1131 | 129 | 111.08 | 0.14 | 0.089 |
| **Grade** |  |  |  |  |  |
| 1 | 1744 | 25 | 30.73 | -0.23 | 0.301 |
| 2 | 5507 | 179 | 193.01 | -0.08 | 0.313 |
| 3 | 3679 | 266 | 250.23 | 0.06 | 0.319 |
| **Pathological stage** |  |  |  |  |  |
| 1 | 4481 | 58 | 60.40 | -0.04 | 0.758 |
| 2 | 4488 | 149 | 154.20 | -0.03 | 0.675 |
| 3 | 1713 | 196 | 191.42 | 0.02 | 0.741 |
| 4 | 248 | 67 | 67.95 | -0.01 | 0.908 |
| **Chemotherapy** |  |  |  |  |  |
| without | 3590 | 134 | 136.71 | -0.02 | 0.817 |
| with | 7340 | 336 | 337.26 | -0.004 | 0.945 |
| **Radiotherapy** |  |  |  |  |  |
| without | 4890 | 255 | 252.75 | 0.01 | 0.888 |
| with | 6040 | 215 | 221.22 | -0.03 | 0.676 |
| **Age at diagnosis** |  |  |  |  |  |
| <35 | 425 | 13 | 14.71 | -0.13 | 0.656 |
| 35 to 49 | 3846 | 109 | 109.32 | -0.003 | 0.976 |
| 50 to 64 | 4861 | 185 | 190.89 | -0.03 | 0.670 |
| 65 to 74 | 1298 | 89 | 87.75 | 0.01 | 0.894 |
| 75+ | 500 | 74 | 71.31 | 0.04 | 0.750 |
| **Tumor size** |  |  |  |  |  |
| <10 | 1406 | 17 | 12.24 | 0.28 | 0.174 |
| 10 to 19 | 3692 | 61 | 68.78 | -0.13 | 0.348 |
| 20 to 29 | 2897 | 105 | 114.88 | -0.09 | 0.357 |
| 30 to 49 | 2072 | 154 | 150.45 | 0.02 | 0.773 |
| 50+ | 863 | 133 | 127.61 | 0.04 | 0.633 |
| **Hormone/steroid therapy** | | | | | |
| without | 2987 | 262 | 241.30 | 0.08 | 0.183 |
| with | 7943 | 208 | 232.68 | -0.12 | 0.106 |
| **Targeted therapy** |  |  |  |  |  |
| without | 9101 | 370 | 374.35 | -0.01 | 0.822 |
| with | 1829 | 100 | 99.62 | 0.004 | 0.969 |
| **Lymph vessels or vascular invasion** | | | | | |
| No | 6894 | 171 | 174.11 | -0.02 | 0.814 |
| Yes | 4036 | 299 | 299.86 | -0.003 | 0.960 |

**Table S20. Model 4 calibration at 3 years in testing data.**

|  | **Number of cases** | **Observed events** | **Predicted events** | **difference (%)** | ***P* value** |
| --- | --- | --- | --- | --- | --- |
| **Subtype** |  |  |  |  |  |
| Luminal-like | 3451 | 101 | 109.53 | -0.08 | 0.415 |
| HER2 | 1412 | 65 | 68.48 | -0.05 | 0.674 |
| Triple negative | 618 | 76 | 61.76 | 0.19 | 0.070 |
| **Grade** |  |  |  |  |  |
| 1 | 909 | 8 | 16.35 | -1.04 | 0.039 |
| 2 | 2771 | 90 | 96.48 | -0.07 | 0.509 |
| 3 | 1801 | 144 | 126.94 | 0.12 | 0.130 |
| **Pathological stage** |  |  |  |  |  |
| 1 | 2258 | 28 | 29.44 | -0.05 | 0.790 |
| 2 | 2260 | 93 | 80.30 | 0.14 | 0.156 |
| 3 | 829 | 78 | 90.28 | -0.16 | 0.196 |
| 4 | 134 | 43 | 39.75 | 0.08 | 0.606 |
| **Chemotherapy** |  |  |  |  |  |
| without | 1812 | 54 | 69.02 | -0.28 | 0.071 |
| with | 3669 | 188 | 170.75 | 0.09 | 0.187 |
| **Radiotherapy** |  |  |  |  |  |
| without | 2429 | 121 | 125.41 | -0.04 | 0.693 |
| with | 3052 | 121 | 114.36 | 0.05 | 0.535 |
| **Age at diagnosis** |  |  |  |  |  |
| <35 | 201 | 13 | 9.29 | 0.29 | 0.224 |
| 35 to 49 | 1935 | 59 | 57.87 | 0.02 | 0.882 |
| 50 to 64 | 2382 | 94 | 91.72 | 0.02 | 0.812 |
| 65 to 74 | 693 | 43 | 44.67 | -0.04 | 0.802 |
| 75+ | 270 | 33 | 36.21 | -0.10 | 0.593 |
| **Tumor size** |  |  |  |  |  |
| <10 | 657 | 4 | 5.69 | -0.42 | 0.479 |
| 10 to 19 | 1945 | 34 | 36.96 | -0.87 | 0.626 |
| 20 to 29 | 1467 | 56 | 62.04 | -0.11 | 0.443 |
| 30 to 49 | 983 | 82 | 72.72 | 0.11 | 0.277 |
| 50+ | 429 | 66 | 62.36 | 0.06 | 0.645 |
| **Hormone/steroid therapy** | | | | | |
| without | 1553 | 139 | 123.52 | 0.11 | 0.164 |
| with | 3928 | 103 | 116.26 | -0.13 | 0.219 |
| **Targeted therapy** |  |  |  |  |  |
| without | 4595 | 197 | 193.78 | 0.02 | 0.817 |
| with | 886 | 45 | 46.00 | -0.02 | 0.883 |
| **Lymph vessels or vascular invasion** | | | | | |
| No | 3485 | 95 | 89.25 | 0.06 | 0.543 |
| Yes | 1996 | 147 | 150.52 | -0.02 | 0.774 |
